# Developmental risk factors for eating disorder symptoms in adolescents: findings from the Australian Temperament Study

**DOI:** 10.1186/2050-2974-2-S1-O44

**Published:** 2014-11-24

**Authors:** Elizabeth K Hughes, Daniel Le Grange, Meredith O'Connor, Jacqui Macdonald, Keri Little, Craig A Olsson

**Affiliations:** 1 University of Melbourne, Melbourne, Australia; 2 Murdoch Childrens Research Institute, Melbourne, Australia; 3 Royal Children's Hospital, Melbourne, Australia; 4 University of Chicago, Chicago, USA; 5 Geelong Grammar School, Melbourne, Australia; 6 Deakin University, Melbourne, Australia

## 

The study used longitudinal data from an Australian population based birth cohort to identify developmental risk factors for eating disorder symptoms in adolescents. The aims were to: (1) develop a confirmatory factor model of risky eating attitudes and behaviours at 15-16 years of age based on the Eating Disorder Inventory (EDI), (2) develop a path model of childhood predictors of adolescent eating disorder symptoms, and (3) examine gender invariance to see whether predictors differed for males and females. Data were drawn from the Australian Temperament Study, a longitudinal study of the health and development of a population-based cohort across 15 waves of data collection from infancy to adulthood. Participants in the current study were the 1,300 youth who completed the EDI at age 15-16 years. Eating disorder symptoms in mid-adolescence was best explained by multiple factors from individual, interpersonal, and family domains. The relative importance of predictors differed for males and females. The findings contribute to our understanding of psychosocial and environmental factors influencing pathways towards disordered eating behaviors in adolescents.

This abstract was presented in the **Prevention & Public Health** stream of the 2014 ANZAED Conference.

